# Validity and reliability of the Mini International Neuropsychiatric Interview in Sub-Saharan Africa: a cross-country comparison study

**DOI:** 10.1017/S0033291725100573

**Published:** 2025-08-29

**Authors:** Kristina J. Korte, Kimberly Hook, Rocky Stroud, Amantia Ametaj, Manasi Sharma, Hayden Mountcastle, Biruh Alemayehu, Beakal Amare, Azeb Asaminew Alemu, Ribka Birhanu, Engida Girma, Barkot Milkias, Mahlet Yared, Florence Jaguga, Jackline Mmochi, Felitcita Omari, Edgar Guma, Hillary Kutessa, Claire Kwagala, Harriet Nakuya, Molly Naisanga, Dickens Akena, Lukoye Atwoli, Symon Kariuki, Charles R. J. C. Newton, Zukiswa Zingela, Dan J. Stein, Teferra Solomon, Karestan C. Koenen, Bizu Gelaye

**Affiliations:** 1 Department of Psychiatry, Massachusetts General Hospital, Boston, MA, USA; 2 Department of Epidemiology, Harvard T.H. Chan School of Public Health, Boston, MA, USA; 3 Stanley Center for Psychiatric Research, Broad Institute of MIT and Harvard, Cambridge, MA, USA; 4 Department of Psychology, Northeastern University, Boston, MA, USA; 5 Department of Psychiatry, Addis Ababa University, Addis Ababa, Ethiopia; 6 Department of Mental Health, Moi Teaching and Referral Hospital, Eldoret, Kenya; 7 Department of Psychiatry, School of Medicine, College of Health Sciences, Makerere University, Kampala, Uganda; 8 Brain and Mind Institute, Medical College East Africa, The Aga Khan University, Nairobi, Kenya; 9 Neurosciences Unit, Clinical Department, KEMRI-Wellcome Trust Research Programme, Kilifi, Kenya; 10 Psychiatry and Behavioural Sciences, Nelson Mandela University, Gqeberha, South Africa; 11 SAMRC Unit on Risk & Resilience in Mental Disorders, Department of Psychiatry and Mental Health, University of Cape Town, Cape Town, South Africa; 12 Epidemiology Branch, Division of Population Health Research, Division of Intramural Research, Eunice Kennedy Shriver National Institute of Child Health and Human Development, Bethesda, MD, USA

## Abstract

**Background:**

Diagnostic tools, such as the Mini International Neuropsychiatric Interview (MINI) 7.0.2 and the Structured Clinical Interview for the DSM-5 (SCID), aim to increase the validity and reliability of diagnostic assessment. However, these tools were created in high-income countries (HICs) with limited investigation of the psychometrics of these tools when used in low- and middle-income countries (LMICs). Thus, there is a need to examine the psychometric properties of these measures in LMICs. The present investigation aimed to examine the use of the MINI in Ethiopia, Kenya, and Uganda.

**Methods:**

A multicountry comparison of the validity and reliability of the MINI was conducted in a study of 954 participants (*n* = 667 cases; *n* = 287 controls) with and without a psychotic spectrum disorder, defined as any psychotic or bipolar spectrum disorder for the NeuroGAP – Psychosis study. Test–retest reliability of the MINI was examined in a subset of 303 participants (*n* = 164 cases; *n* = 139 controls) from the overall sample.

**Results:**

Results revealed the MINI and SCID provided excellent diagnostic accuracy with area under the curve (AUC) values of .91 (*SE* = .01) for the MINI and .95 (*SE* = .01) for the SCID. Positive predictive values (PPV) were the highest for the SCID (93.8%) and slightly lower for the MINI (88.7%). Reliability analyses revealed substantial agreement for psychotic and bipolar diagnostic groups.

**Conclusions:**

Similar patterns of results were observed at the country level with a few notable differences. Limitations and future directions are discussed.

## Introduction

Psychotic and bipolar spectrum disorders are associated with significant distress and burden across the globe (Chong et al., 2016; De Oliveira, Cheng, Rehm, & Kurdyak, 2016; Teigland, Mohammadi, Agatep, Boskovic, & Sajatovic, 2024; Woods, 2000), making the reliable and valid assessment of these conditions paramount. There are numerous measures created to assess for psychotic disorders, ranging from screening for psychosis (e.g., Psychotic Screening Questionnaire (PSQ; Bebbington & Nayani, 1995), self-report assessment of psychotic symptoms (e.g., Positive and Negative Symptoms Questionnaire; PNS-Q; Iancu, Poreh, Lehman, Shamir, & Kotler, 2005) to the use of diagnostic interviews, such as the Mini International Neuropsychiatric interview 7.0.2 (MINI; Lecrubier et al., 1997; Sheehan et al., 1998).

Valid and reliable assessment tools are critical in clinical investigations to ensure a valid and reliable diagnosis of mental health conditions. Several structured and semi-structured clinical interviews have been developed to provide a standardized approach to improve the validity and reliability of diagnostic assessment. The MINI is used across the globe (Mordal, Gunderson, & Bramness, 2010; Otsubo et al., 2005; Rossi et al., 2004; Sharifi et al., 2009); however, few studies have evaluated the psychometric properties of these measures in diverse settings, such as in sub-Saharan Africa. Thus, there is a need to assess the psychometric properties of these measures in these regions to ensure a valid and reliable assessment of mental health conditions across the globe.

Structured interviews such as the MINI offer several advantages for assessing psychotic and bipolar disorders over self-report questionnaires. A key strength of these measures is that they can be both clinician and professional interviewer-administered measures designed to improve the validity and reliability of diagnosing mental health disorders over the use of self-report measures or nonstructured diagnostic approaches. The fully structured nature of the MINI reduces the need for prompting and follow-up questions that an interviewer needs to ask to reach a diagnosis. In prior studies, MINI was found to have sound psychometric properties and to be a valid and reliable measure in a variety of countries and contexts (Mordal, Genderson, & Bramness, 2010; Otsubo et al., 2005; Rossi et al., 2004; Sharifi et al., 2009), including some LMICs (Chellamuthu et al., 2017; Huang, Liu, Wang, et al., 2016). Despite these findings, this area of research is limited in that existing studies are overwhelmingly focused on the Global North, thereby raising the question of how these measures perform in diverse settings and contexts, such as sub-Saharan Africa.

To the best of our knowledge, our group is the only group that has examined the diagnostic validity of the MINI in sub-Saharan Africa. In two prior studies, we examined other aspects of validity, primarily construct validity. In Kenya, we (Jaguga et al., 2022) performed a multigroup (e.g., sex) comparison of item response and the latent structure of the MINI psychosis items. Item response differences were observed in males and females; however, the factor structure was invariant across groups, suggesting there were no sex-related differences in the factor structures of the MINI psychosis items. Subsequently, we (Korte et al., 2023) took this investigation a step further by examining the differential item response of the MINI psychosis items across four countries in sub-Saharan Africa (Ethiopia, Kenya, South Africa, and Uganda; Korte et al., 2023). Differences in item responses were observed across the countries, suggesting that different items in the MINI psychotic model predicted being high or low on the latent construct of psychosis (e.g., being high on auditory hallucinations (i.e., MINI item K6) was predictive of being high on psychosis in South Africa; whereas being high on odd beliefs (i.e., MINI item K5) was more predictive of psychosis in Kenya). These studies provided preliminary findings of the psychometric properties of the MINI psychosis items related to the factor structure and item-level functioning; they did not examine the validity and reliability of the MINI.

The aim of the current study was to examine the reliability and validity of the MINI in a large sample (*N* = 954) of participants with psychotic and bipolar spectrum disorders from three countries in sub-Saharan Africa (i.e., Ethiopia, Kenya, and Uganda). Specifically, we examined the criterion validity of the MINI compared to the SCID for the overall sample and by country. Given the research supporting the notion that psychotic and bipolar disorders fall along the same continuum (Shelvin et al., 2017), we also examined whether diagnostic accuracy would be higher in the combined bipolar and psychotic disorder groups compared to the separate psychotic disorder and bipolar disorder groups in the MINI. Further, test–retest reliability was assessed in a subset of the overall sample. It was predicted that the MINI would demonstrate acceptable validity and that the MINI would demonstrate acceptable test–reretest reliability.

## Methods

Data for this manuscript were collected as part of a larger parent study (Neuropsychiatric Genetics of African Populations – Psychosis; “NeuroGAP-Psychosis”; NeuroGAP – Psychosis), which was a case–control study spanning 2018–2023 that collected data to identify genetic and environmental risk factors associated with psychotic disorders in Ethiopia, Kenya, South Africa, and Uganda. The data used for the present study comprised two samples – (1) a validity sample used to assess the validity of the MINI (*n* = 954) and (2) a reliability sample (*n* = 303) comprised of a subset of the validity sample that returned for the reliability portion of the study. This study was conducted in the Ethiopian, Kenyan, and Ugandan sites of the NeuroGAP-Psychosis parent study. Full details of the study are described below.

*Participants.* For the criterion validity sample, participants were comprised of 954 participants from Ethiopia (*n* = 314), Kenya (*n* = 326), and Uganda (*n* = 314). The breakdown of cases and controls by country is 223 cases and 91 controls in Ethiopia, 222 cases and 104 controls in Kenya, and 222 cases and 92 controls in Uganda. A majority of the participants were male (54.6%) with a mean age of 53.4 (*SD* = 11.0). For the reliability sample, a subset of 303 participants (*n* = 164 cases, *n* = 139 controls) from the validity study completed the reliability portion of the study (*n* = 100 in Ethiopia, 103 in Kenya, and 100 in Uganda). See Table 1 for participant characteristics for the overall sample by country and by cases and controls.

**Table 1.** Participant characteristics for the full sample and by country

	All sites	Ethiopia	Kenya	Uganda
*N*	954	314	326	314
Age (years), mean (SD)	35.4 (11.0)	35.2 (10.1)	35.6 (11.4)	35.4 (11.4)
Sex (%)				
Male	502 (52.6%)	191 (60.8%)	155 (47.5%)	156 (49.7%)
Female	452 (47.4%)	123 (39.2)	171 (52.5%)	158 (50.3%)
Educational status (%)				
No education/some primary school	206 (21.6%)	78 (24.8%)	29 (8.9%)	99 (31.5%)
Finished primary/some secondary school	349 (36.6%)	86 (27.4%)	114 (35.0%)	149 (47.5%)
Finished secondary/some college	222 (23.3%)	87 (27.7%)	94 (28.8%)	41 (13.1%)
Finished college or beyond	177 (18.6%)	63 (20.1%)	89 (27.3%)	25 (8.0%)
Living arrangement (%)				
Lives alone	118 (12.4%)	51 (16.2%)	43 (13.2%)	24 (7.6%)
Lives with parental family	279 (29.2%)	133 (42.4%)	113 (34.7%)	33 (10.5%)
Lives with spouse or partner	276 (28.9%)	93 (29.6%)	124 (38.0%)	59 (18.8%)
Lives with other relatives and/or friends	281 (29.5%)	37 (11.8%)	46 (14.1%)	198 (63.1%)
Marital status (%)				
Married or cohabitating	324 (34.0%)	96 (30.6%)	129 (39.6%)	99 (31.5%)
Widowed, divorced, separated	214 (22.4%)	44 (14.0%)	55 (16.9%)	115 (36.6%)
Never married	416 (43.6%)	174 (55.4%)	142 (43.6%)	100 (31.8%)
Language of consent (%)				
Amharic	314 (32.9%)	314 (100%)	–	–
Kiswahili	326 (34.2%)	–	326 (100%)	–
Luganda	314 (32.9%)	–	–	314 (100%)

*Note: N* = 954 for the full sample, *n* = 314 Ethiopia, *n* = 326 for Kenya, and *n* = 314 for Uganda.

Inclusion and exclusion criteria for the cases and control status were determined during the baseline assessment of the NeuroGAP – Psychosis parent study and are described in detail elsewhere (see Stevenson et al., 2019). Briefly, to be eligible for the study, cases were required to have a clinical diagnosis of psychosis (i.e., schizophrenia, schizoaffective disorder, psychotic disorder not otherwise specified, and bipolar disorder) as assessed by a chart review. Controls were participants without a psychotic disorder diagnosis. Both cases and control participants were excluded if they were under the age of 18 years old, were acutely psychotic, had severe alcohol or substance misuse as defined by being inpatient or under medical care for a substance use disorder, not fluent in one of the study languages, or unable to provide consent as assessed by the University of California, San Diego Brief Assessment of Capacity to Consent (UBACC; Jeste et al., 2007).

### Measures

The present study utilized a demographic questionnaire, the MINI, and the SCID. The use of the MINI and SCID was guided by the need to balance thoroughness and practicality for the present study. The MINI allows for quicker assessments while maintaining a reasonable level of diagnostic accuracy, which is crucial in resource-limited settings. Conversely, the SCID, though more time-intensive, offers a high standard for diagnosis and provides an optimal comparison to validate the results obtained from the MINI.

Demographic variables of interest were gathered using a demographic questionnaire that was developed by research staff for the NeuroGAP- Psychosis parent study. The demographic questionnaire assessed age, sex, education, marital status, and other demographic variables relevant to the parent study.

*The Mini International Neuropsychiatric Interview, Standard 7.0.2 for Diagnostic and Statistical Manual of Mental Disorders-5 (MINI).* The MINI (Lecrubier et al., 1997; Sheehan et al., 1998) was used to assess for mental health diagnoses. The MINI is a structured interview assessing the presence of current and past mental health disorders based on diagnostic criteria from *the Diagnostic and Statistical Manual* (*DSM*-5; American Psychiatric Association, 2013) and the *International Classification of Diseas*e (ICD-10; World Health Organization, 2019). Participants completed modules A, C, K, and O of the MINI (Sheehan et al., 1998), which evaluated major depressive episodes, manic and hypomanic episodes, psychotic disorders, and mood disorders with psychotic features, respectively.

The *Structured Clinical Interview for the DSM-5* (SCID; First, Williams, Karg, & Spitzer, 2015) was used to assess the validity of the MINI. The SCID is a semi-structured interview assessing mental health diagnoses from the DSM-5 (APA, 2013). SCID modules A (mood disorders), B (psychotic disorders), C (psychotic disorder differential), and D (mood disorder differential).

### Procedure

Participants for the validity and reliability study were recruited from the NeuroGAP-Psychosis parent study. For the parent study, cases were recruited from outpatient clinical sites in Ethiopia, Kenya, and Uganda. Participants were also recruited from inpatient settings in Ethiopia. Control participants were recruited from general medical facilities and were comprised of students, family members accompanying individuals to medical appointments, and other people presenting at the general medical clinics (see Stevenson et al., 2019 for full details).

Cases and controls who were enrolled in the parent study were invited to join the validity and reliability study. Participants were administered the MINI as part of their baseline assessment for the NeuroGAP – Psychosis parent study. For those who agreed to participate in the validity and reliability study, a psychiatrist administered the SCID diagnostic interview immediately upon completion of the baseline assessment for the parent study. Upon completion of the SCID interview, a subgroup of the validity participants was recruited to complete the reliability study. Participants agreeing to the reliability study returned 2 weeks after completing the baseline assessment (when the MINI was first administered) and were administered the MINI a second time by a different interviewer to allow for an assessment of the test–retest reliability of the MINI.

*Training and Supervision.* Local research staff were trained to conduct structured and semi-structured diagnostic interviews using the MINI and SCID. Given that the MINI is a structured diagnostic interview that can be administered by trained nonclinicians, the MINI was administered by the NeuroGAP – Psychosis study research staff with a range of training backgrounds (e.g., psychiatrists, masters, and bachelor-level research staff). Those administering the MINI received in-person training from the developer of the MINI before participants enrolled in the parent study. In contrast, the SCID is a semi-structured interview requiring specialized clinical training by mental health professionals. To test the validity of the MINI compared to the SCID, local psychiatrists received formal training conducted by doctoral-level psychologists. Training occurred over 1-week in-person training workshops and was ongoing with virtual refresher sessions via Zoom due to the COVID-19 pandemic. Rigorous supervision was continuous throughout the study, such that every SCID was reviewed item-by-item by psychologists and reviewed with the local team in weekly supervision meetings. The function of these weekly supervision meetings was to ensure accurate diagnoses, agreed upon by multiple doctoral-level mental health professionals, as well as to ensure data accuracy. Errors were reviewed each week with the research staff and corrected to minimize errors.

*Ethics.* The NeuroGAP-Psychosis study was approved by the Institutional Review Board at Harvard T.H Chan School of Public Health (#IRB17–0822) in the USA and the following ethics committees: Addis Ababa University College of Health Sciences (#014/17/Psy) and the Ministry of Science and Technology Ethics Committee (#3.10/14/2018), Moi Teaching and Referral Hospital Ethics Committee (#IREC/2016/145), Kenya National Council of Science and Technology (#NACOSTI/P/17/56302/19576) KEMRI Centre Scientific Committee (CSC# KEMRI/CGMRC/CSC/070/2016), KEMRI Scientific and Ethics Review Unit (SERU#KEMRI/SERU/CGMR-C/070/3575); The Makerere University School of Medicine (SOMREC #REC REF 2016–057), and the Uganda National Council for Science and Technology Ethics Committee (UNCST #HS14ES).

### Data analytic plan

All analyses were conducted using IBM SPSS Statistics 29. Receiver operating curve (ROC) analysis was used to evaluate the area under the curve (AUC), specificity, and sensitivity of the MINI and the SCID. ROC analyses were run to assess: (1) chart diagnosis versus MINI diagnosis, (2) chart diagnosis versus SCID diagnosis, and (3) SCID as reference versus MINI diagnosis. Binary logistic regression was used to compute positive predictive values (PPV) for the MINI and the SCID. Kappa reliability coefficients were computed to compare the MINI diagnoses at baseline in comparison to the MINI diagnoses at the 2-week follow-up.

*Grouping variables.* Since the version of the MINI used in the present study only provides a general diagnosis of “psychotic disorder,” we computed grouping variables to allow for comparisons with the SCID, which provides a more nuanced diagnostic approach, allowing for diagnosis of the full range of psychotic spectrum disorders (e.g., schizophrenia). Specifically, we created six composite diagnostic groups (three groups for the MINI and three groups for the SCID) for the MINI and SCID comprised of: (1) “Any Psychotic Disorder,” (2) “Any Bipolar Disorder,” and (3) a composite group “Any NeuroGAP Psychosis Case,” defined as having any psychotic or bipolar disorder as used in the NeuroGAP parent study. For the MINI, the “Any Psychotic Disorder” group consists of the psychotic disorder variable on the MINI. The “Any Bipolar Disorder Group” on the MINI is comprised of bipolar I, bipolar II, and other bipolar disorder variables on the MINI. The “Any NeuroGAP Psychosis Case” group for the MINI is a composite of the “Any Psychotic Disorder” and “Any Bipolar Disorder” groups. For the SCID, the “Any Psychotic Disorder” group was comprised of the schizophrenia, schizoaffective, schizophreniform, and other specified psychotic disorder variables on the SCID. The SCID “any bipolar” group was comprised of the SCID bipolar disorder I, bipolar disorder II, and other bipolar disorder variables. The “Any NeuroGAP Psychosis Case” group for the SCID was a combination of the aforementioned SCID psychosis and bipolar variables. For both the MINI and SCID, group assignment was based on the presence (coded 1) or absence (coded 0) of one or more of the diagnoses in the group. The use of these grouping variables also allows for an exploration of the psychosis and bipolar continuum (Shelvin et al., 2017) by comparing the diagnostic specificity of the composite Any NeuroGAP Psychosis Case group with the Any Psychotic Disorder and Any Bipolar Disorder groups. Analyses were conducted for all of the computed grouping variables (Any Psychotic Disorder, Any Bipolar Disorder, and Any NeuroGAP Psychosis Case (any diagnosis of a psychotic or bipolar disorder) as defined by the eligibility criteria for the NeuroGAP Psychosis study. All analyses were conducted on the full sample and then by country.

## Results

*Symptom level endorsement.* Endorsement of psychotic symptoms in cases in the full sample was high on the MINI and SCID. Percent endorsement of psychotic symptoms on the MINI-7 ranged from 80.2% for item K5 “odd beliefs” to 46.8% for item K4 “delusions of reference.” The endorsement was also high in the SCID and ranged from 60.7% for item B16 “auditory hallucinations” and a low of 0.3% on item B32 “stereotypy” on the SCID. Symptom endorsement for manic symptoms was high on the MINI, ranging from 81.9% for item C3d measuring “rapid thoughts” to 45.3% for item C3g “high-risk behaviors.” However, the endorsement was lower on the SCID, with a range of 23.4% for item A92 “manic mood” to 12.1% for item A104 “high-risk behaviors.” See Table 2 for endorsement of all symptoms of psychosis and bipolar disorder on the MINI and SCID for the full sample.

**Table 2.** MINI and SCID-5 lifetime diagnoses of psychotic and bipolar disorder

Measure					
MINI-7			SCID-5		
Dx	Cases	Controls	Dx	Cases	Controls
Full sample					
Any NGAP psychosis case	82.0% (547)	1.7% (5)	Any NGAP psychosis case	92.2% (615)	2.4% (7)
Any psychotic disorder	43.8% (292)	1.7% (5)	Any psychotic disorder	63.6% (424)	2.1% (6)
Any bipolar disorder	58.5% (390)	.7% (2)	Any bipolar disorder	29.1% (194)	.3% (1)
Ethiopia					
Any NGAP psychosis case	76.1% (169)	0% (0)	Any NGAP psychosis case	93.7% (208)	0% (0)
Any psychotic disorder	46.8% (104)	0% (0)	Any psychotic disorder	73.4 (163)	0% (0)
Any bipolar disorder	50.9% (113)	0% (0)	Any bipolar disorder	20.7% (46)	0% (0)
Kenya					
Any NGAP psychosis case	83.3% (185)	0% (0)	Any NGAP psychosis case	91.4% (204)	0% (0)
Any psychotic disorder	5% (11)	0% (0)	Any psychotic disorder	52.7% (117)	0% (0)
Any bipolar disorder	84.7% (188)	0% (0)	Any bipolar disorder	38.7% (86)	0% (0)
Uganda					
Any NGAP psychosis case	86.5% (193)	5.5% (5)	Any NGAP psychosis case	91.5% (204)	7.7% (7)
Any psychotic disorder	79.4% (177)	5.5% (5)	Any psychotic disorder	64.6% (144)	6.6% (6)
Any bipolar disorder	39.9% (89)	2.2% (2)	Any bipolar disorder	27.8% (62)	1.1% (1)

*Note: N* = 954 for the full sample, *n* = 314 Ethiopia, *n* = 326 for Kenya, and *n* = 314 for Uganda. Categories are not mutually exclusive and participants could be assigned to more than one group depending on comorbid diagnoses.

*Diagnostic specificity.* ROC analyses were conducted to assess diagnostic specificity for: (1) Any NeuroGAP Psychosis Case, (2) Any Psychotic Disorder, and (3) Any Bipolar Disorder groups on the MINI and SCID. Chart diagnosis (i.e., 0 = no diagnosis, 1 = any psychotic or bipolar disorder) was entered as the reference group to provide the diagnostic comparison with the MINI (see Table 3). Finally, we also examined the diagnostic specificity analyses examining the ROCs of the MINI-7 with the SCID as the reference group (see Table 4) to provide a direct comparison of the diagnostic specificity of the MINI compared to the SCID. AUC values range from 0 to 1.0, with values less than .50 indicating no discrimination, .70 to .80 acceptable discrimination, .80 to .90 reflecting excellent discrimination, and values .90 and great resulting in outstanding discrimination (Safari, Baratloo, Elfil, & Negida, 2016). Finally, PPVs for cases, controls, and the combined sample were computed for the MINI and SCID. Analyses were conducted on the full sample and by country (i.e., Ethiopia, Kenya, and Uganda).

**Table 3.** Item level comparisons of the MINI-7 and the SCID item level endorsements of psychotic and bipolar disorder symptoms

Symptoms	MINI item	% endorsement (*N*)	SCID item	% endorsement (*N*)
Delusions	K1 – Persecutory delusions	77.1% (514)	B2 – Persecutory	46.8% (312)
	K2 – Thought broadcasting	61.0% (407)	B12 – Thought broadcasting	9.8% (65)
	K3- Thought insertion	57.4% (383)	B10 Thought insertion	10.5% (70)
	K4 – Reference	46.2% (308)	B1 – Reference	36.8% (245)
	K5 – Odd beliefs	80.2% (535)	B14 – Bizarre	14.6% (97)
	–	–	B11 – Thought removal	9.6% (64)
	–	–	B3 – Grandiose	25.9% (173)
	–	–	B4 – Somatic	4% (27)
	–	–	B5 – Delusion of Guilt	4.1% (27)
	–	–	B6 – Jealous	3.3% (22)
	–	–	B7 – Erotomatic	1.6% (11)
	–	–	B8 – Religious	19% (127)
	–	–	B9 – Being controlled	23.5% (157)
	–	–	B13 – Other	8.4% (56)
Hallucinations	K6 – Auditory	78.9% (526)	B16- Auditory	60.7% (404)
	K7 – Visual	46.8% (312)	B17 – Visual	33.7% (225)
	–	–	B18 – Tactile	9.1% (61)
	–	–	B19 – Somatic	6.2% (41)
	–	–	B20 – Gustatory	1.9% (13)
	–	–	B21 – Olfactory	4.7% (31)
Disorganized speech	K8 – Disorganized speech	69.0% (460)	B24 – Disorganized speech	18.4% (123)
Disorganized/catatonic behavior	K9 – Disorganized/catatonia	53.8% (359)	B26 – Disorganized behavior	13.3% (89)
	–	–	B27 – Stupor	0.6% (4)
	–	–	B28 – Grimacing	0.7% (5)
	–	–	B29 – Mannerism	0.9% (6)
	–	–	B30 – Posturing	1.2% (8)
	–	–	B31 – Agitation	2.5% (17)
	–	–	B32 – Stereotypy	0.3% (2)
	–	–	B33 – Mutism	1.9% (13)
Negative Symptoms	K10 – Negative symptoms	50.2% (335)	–	–
	–	–	B42 – Diminished expressiveness	16.9% (113)
Depression	A1 – Low mood	58.2% (388)	A27 – Low mood	18.1% (121)
	A2 – Anhedonia	56.5% (377)	A28 – Anhedonia	17.3% (115)
	A3a – Change in appetite	86.1% (358)	A29 – Change in appetite	14.8% (99)
	A3b – Sleep disturbance	90.9% (378)	A32 – Sleep disturbance	16.3% (109)
	A3c – Psychomotor retardation	82.2% (342)	A35 – Psychomotor retardation / agitation	13.8% (92)
	A3d – Low energy	71.6% (298)	A38 – Low energy	13.3% (89)
	A3e – Worthless/guilt	73.3% (305)	A39 – Worthless/guilt	14.4% (96)
	A3f – Trouble concentrating	85.1% (354)	A42 – Trouble concentrating	13.8% (92)
	A3g – Suicidal thoughts	50.2% (209)	A43 – Suicidal thoughts	11.7% (78)
Mania	–	–	A92 – Manic mood	23.4% (156)
	C1 – Elevated mood	54.3% (362)	A93 – Elevated mood	13.2% (88)
	C2 – Irritable mood	59.2% (395)	A94 – Irritable mood	12.7% (85)
	C3a – Grandiosity	67.9% (304)	A96 – Grandiosity	15.6% (104)
	C3b – Reduced sleep	81.2% (364)	A97 – Reduced sleep	21.1% (141)
	C3c – Pressured speech	80.4% (360)	A98 – Pressured speech	19.9% (133)
	C3d – Rapid thoughts	81.9% (367)	A99 – Racing thoughts	19.6% (131)
	C3e – Easily distracted	78.8% (353)	A100 – Easily distracted	15.9% (106)
	C3f – Increased activity	77.2% (346)	A101 – Increased activity	20.1% (134)
	C3g – High-risk behaviors	45.3% (203)	A104 – High-risk behaviors	12.1% (81)

**Table 4.** Diagnostic specificity of the MINI and SCID for the Full sample and by country

*Chart versus MINI							
Country	Diagnostic group	PPV cases	PPV controls	PPV total	Sensitivity	1-Specificity	AUC (SE)
Full sample	Any NGAP case	84.1%	98.3%	88.7%	0.84	0.01	.91 (.01)
	Any psychotic Dx	100.0%	0.0%	70.0%	0.44	0.02	.71 (.02)
	Any bipolar Dx	58.5%	99.3%	70.7%	0.59	0.01	.79 (.01)
Ethiopia	Any NGAP case	78.8%	100.0%	85.0%	0.79	0.00	.89 (.02)
	Any psychotic Dx	100.0%	0.0%	70.7%	0.47	0.00	.73 (.03)
	Any bipolar Dx	100.0%	0.0%	70.7%	0.51	0.00	.76 (.03)
Kenya	Any NGAP case	85.6%	100.0%	90.2%	0.86	0.00	.93 (.02)
	Any psychotic Dx	100.0%	0.0%	68.1%	0.05	0.00	.53 (.03)
	Any bipolar Dx	84.7%	100.0%	89.6%	0.85	0.00	.92 (.02)
Uganda	Any NGAP case	87.9%	94.4%	89.8%	0.88	0.06	.91 (.02)
	Any psychotic Dx	79.4%	94.4%	83.7%	0.79	0.06	.87 (.02)
	Any bipolar Dx	100.0%	0.0%	71.2%	0.40	0.02	.69 (.03)

*Chart versus SCID							
Country	Diagnostic group	PPV cases	PPV controls	PPV total	Sensitivity	1-Specificity	AUC (SE)
Full sample	Any NGAP case	92.2%	97.6%	93.8%	0.92	0.02	.95 (.01)
	Any psychotic Dx	63.6%	97.9%	73.9%	0.64	0.01	.81 (.01)
	Any bipolar Dx	100.0%	0.0%	69.9%	0.29	0.01	.65 (.03)
Ethiopia	Any NGAP case	93.7%	100.0%	95.5%	0.94	0.00	.97 (.01)
	Any psychotic Dx	73.4%	100.0%	81.2%	0.73	0.00	.87 (.02)
	Any bipolar Dx	100.0%	0.0%	70.7%	0.21	0.00	.60 (.03)
Kenya	Any NGAP case	91.4%	100.0%	94.2%	0.91	0.00	.96 (.01)
	Any psychotic Dx	100.0%	0.0%	68.1%	0.53	0.00	.76 (.03)
	Any bipolar Dx	100.0%	0.0%	68.1%	0.39	0.00	.69 (.03)
Uganda	Any NGAP case	91.5%	92.3%	91.7%	0.92	0.08	.92 (.02)
	Any psychotic Dx	64.6%	93.4%	72.9%	0.65	0.07	.79 (.03)
	Any bipolar Dx	100.0%	0.0%	71.0%	0.28	0.01	.64 (.03)

*SCID versus MINI							
Country	Diagnostic group	PPV cases	PPV controls	PPV total	Sensitivity	1-Specificity	AUC (SE)
Full sample	Any NGAP case	84.1%	87.7%	85.6%	0.85	0.12	.86 (.01)
	Any psychotic Dx	84.2%	50.0%	68.7%	0.5	0.16	.67 (.02)
	Any bipolar Dx	100.0%	0.0%	79.5%	0.79	0.32	.74 (.02)
Ethiopia	Any NGAP case	79.3%	91.0%	83.1%	0.79	0.09	0.85 (.02)
	Any psychotic Dx	52.8%	88.1%	69.7%	0.53	0.12	.70 (.03)
	Any bipolar Dx	52.8%	88.0%	69.7%	0.66	0.31	.66 (.04)
Kenya	Any NGAP case	85.7%	87.0%	86.2%	0.86	0.13	.86 (.02)
	Any psychotic Dx	0.0%	100.0%	64.1%	0.03	0.03	.50 (.03)
	Any bipolar Dx	0.0%	100.0%	73.6%	0.94	0.45	.75 (.03)
Uganda	Any NGAP case	88.8%	85.4%	87.5%	0.89	0.15	.87 (.02)
	Any psychotic Dx	83.2%	64.6%	73.5%	0.83	0.35	.74 (.03)
	Any bipolar Dx	0.0%	100.0%	79.9%	0.68	0.19	.75 (.04)

*Note: N* = 954 for the full sample, *n* = 314 Ethiopia, *n* = 326 for Kenya, and *n* = 314 for Uganda. * = reference group for the analyses. AUC = area under the curve. PPV = positive predictive values, SE = Standard error, NGAP = NeuroGAP, Dx = diagnosis.

*Diagnostic Accuracy of the MINI.* First, we examined the diagnostic accuracy of the MINI-7 for the Any NeuroGAP Psychosis Case, Any Psychotic Disorder, and the Any Bipolar Disorder groups using case status as the reference group. The ROC AUC values for the Any NeuroGAP Psychosis Case group on the MINI-7 were .91 (*SE* = .01; sensitivity = .84; PPV = 88.7%, .71 (*SE* = .02; sensitivity = .44; PPV = 70.0%) for the Any Psychotic Disorder group, and .71 (*SE* = .02; sensitivity = .59; PPV = 70.70%) for the Any Bipolar Disorder Group.

At the country level, MINI ROC AUC values for Ethiopia were .89 (*SE* = .02, sensitivity = .79; PPV = 85.00%) for the Any NeuroGAP Psychosis Case group, .73 (*SE* = .03; sensitivity = .47, PPV = 70.70%) for the Any Psychotic Disorder group, and .76 (*SE* = .03; sensitivity = .51, PPV = 70.70%) for Any Bipolar Disorder group. In Kenya, ROC AUC values were .93 (SE = .02; sensitivity = .85; PPV = 90.2%) for the Any NeuroGAP Psychosis Case group, .53 (SE = .03; sensitivity = .05; PPV = 68.10%) for the Any Psychotic Disorder group, and .92 (*SE* = .02; specificity = .85; PPV = 89.60%) for the Any Bipolar Disorder group. In Uganda, ROC AUC values were .91 (SE = .02; sensitivity = .88; PPV = 89.8%) for the Any NeuroGAP Psychosis Case group, .87 (*SE* = .02; sensitivity = .79; PPV = 83.7%) for the Any Psychotic Disorder group, and .69 (*SE* = .03; specificity = .40; PPV = 71.20%) for the Any Bipolar group.

*Diagnostic specificity of the SCID.* Next, we examined the diagnostic specificity of the SCID for the Any NeuroGAP Psychosis Case, Any Psychotic Disorder, and the Any Bipolar Disorder groups for the full sample with case status as the reference group. The ROC AUC values on the SCID were .95 (*SE* = .01; sensitivity .92; PPV = 93.8%) for the Any NeuroGAP Psychosis Case group, .81 (*SE* = .01; sensitivity = .64, PPV = 73.9%) for the Any Psychotic Disorder group, and .65 (SE = .03; sensitivity = .29; PPV = 69.9%) for the Any Bipolar disorder group.

At the country level, SCID ROC AUC values for Ethiopia were .97 (SE = .01; sensitivity = .94; PPV = 95.60%) for the Any NeuroGAP Psychosis Case group, .87 (SE = .02; sensitivity = .73; PPV = 81.20%) for the Any Psychotic Disorder group, and .60 (SE = .03; specificity = .21; PPV = 70.70%) for the Any Bipolar group. In Kenya, ROC AUC values were .96 (SE = .01; sensitivity = .91; PPV = 94.20%) for the Any NeuroGAP Case group, .76 (SE = .03; sensitivity = .53; PPV = 68.10%) for the Any Psychotic Disorder group, and .69 (SE = .02; specificity = .39; PPV = 68.1%) for the Any Bipolar Disorder group. In Uganda, ROC AUC values were .92 (SE = .02; sensitivity = .92; PPV = 91.70%) for the Any NeuroGAP Psychosis Case group, .79 (SE = .03; sensitivity = .65; PPV = 72.90%) for the Any Psychotic Disorder group, and .64 (SE = .03; specificity = .28; PPV = 71.00%) for the Any Bipolar Disorder group. See Table 4 for complete ROC AUC sensitivity results for the full sample and by country and PPV’s by case status (control vs cases).

*Direct comparison of the diagnostic accuracy of the MINI with the SCID.* We also conducted additional analyses examining the ROCs and PPVs of SCID and MINI diagnoses, using the SCID diagnosis as the reference group, to provide a direct comparison of diagnostic concordance of the MINI compared to the SCID. For the full sample, and by country, can be found in Table 4.

*Test–retest reliability of the MINI.* We ran a series of analyses computing Cohen’s kappa reliability coefficients (Cohen, 1960; Galton, 1984) for the full sample, by country, and by case status. Cohen’s kappa reliability coefficient values range from −1 to +1. In the context of test*–*retest reliability, Cohen’s kappa coefficient values less than 0 indicate “no agreement,” .01 to .20 indicate “no to slight agreement,” .21 to .40 reflect “fair” agreement, .41 to .60 indicate “moderate” agreement, .61 to .80 indicating substantial agreement, and .81 to 1.00 reflect “almost perfect agreement” (McHugh, 2012). Kappa values above .60 are viewed to be consistent with good reliability (Cohen, 1960); however, this cutoff is arbitrary, and in diagnostic testing, kappa values of greater than .80 may be more acceptable (McHugh, 2012).

Overall, the test–retest values were good for the overall sample with some country-level differences ranging from good to poor reliability coefficients. The test–retest value for the full sample was .78 (*SE* = .04) for the Any NeuroGAP Psychosis Case group and .62 (*SE* = .05) for the Any Psychotic Disorder and the Any Bipolar Disorder groups, reflecting substantial agreement for all groups in the full sample. At the country level, an interesting pattern emerges, with kappa values ranging from almost perfect agreement to very low, indicating no agreement. In Ethiopia, kappa values were .67 (*SE* = .08) for the Any NeuroGAP Psychosis Case group, .50 (*SE* = .09) for the Any Psychotic Disorder group, and .42 (*SE* = .10) for the Any Bipolar Disorder group, reflecting substantial to moderate agreement across the three groups. In Kenya, kappa values were .82 (*SE* = .06) for the Any NeuroGAP Psychosis Case group, and −.06 (*SE* = .02) for the Any Psychotic Disorder group.86 (*SE* = .06) for the Any Bipolar Disorder group, indicating almost perfect agreement for the Any Bipolar Disorder group to no agreement for the Any Psychotic Disorder group. In Uganda, kappa values were .84 (*SE* = .06) for the Any NeuroGAP Psychosis Case group, .79 (*SE* = .06) for the Any Psychotic Disorder group, and .37 (*SE* = .11) for the Any Bipolar Disorder group, reflecting almost perfect agreement for the Any NeuroGAP Psychosis Case group to fair agreement for the Any Bipolar Disorder group. See Table 5 for the full report of kappa reliability coefficients for the full sample and by country.

**Table 5.** Test–retest reliability of the MINI-7 for the full reliability sample and by country

Country	Diagnostic group	Kappa (SE)
Full sample	Any NGAP case	.78 (.04)
	Any psychotic Dx	.62 (.05)
	Any bipolar Dx	.62 (.05)
Ethiopia	Any NGAP case	.67 (.08)
	Any psychotic Dx	.50 (.09)
	Any bipolar Dx	.42 (.10)
Kenya	Any NGAP case	.83 (.06)
	Any psychotic Dx	−.06 (.02)
	Any bipolar Dx	.86 (.06)
Uganda	Any NGAP case	.84 (.06)
	Any psychotic Dx	.79 (.06)
	Any bipolar Dx	.37 (.11)

*Note. N* = 303; Ethiopia *n* = 100, Kenya *n* = 103, Uganda *n* = 100. *p* > .0001. NGAP = NeuroGAP. Dx = diagnosis.

## Discussion

The present study demonstrated the validity and reliability of the MINI in three countries in sub-Saharan Africa. Overall, the results provide evidence for the criterion validity of the MINI. The pattern of results demonstrates that, in general, diagnostic specificity is higher for the SCID than the MINI across most of the diagnostic grouping variables. This was observed for the overall sample, with some differences when looking at country-level analyses. Specifically, for the full sample, SCID has better diagnostic prediction than MINI for the Any NeuroGAP Psychosis Case and the Any Psychotic Disorder groups; however, MINI has better diagnostic specificity than SCID for the Any Bipolar Disorder group. At the country level, the MINI had better diagnostic specificity of the Any Bipolar Disorder group than the SCID, and the diagnostic specificity for the Any Bipolar Disorder group was higher for the MINI and SCID in Kenya (i.e., .92 and .69, respectively; see Table 4). In contrast, in Ethiopia, the diagnostic specificity for the Any NeuroGAP Psychosis Case and the Any Psychotic Disorder groups was highest when using the SCID. In Uganda, the SCID outperformed the MINI for the Any NeuroGAP Psychosis Case group, albeit with a minuscule difference observed (i.e., .92 and 91, respectively). However, in Uganda, the MINI had higher diagnostic specificity than the SCID for the Any Psychotic Disorder group (i.e., .87 and .79, respectively). When looking at the diagnostic specificity of both the MINI and SCID for all of the disorder groups in the overall sample by country, we see that the diagnostic specificity is higher for the Any NeuroGAP Psychosis Case and Any Psychotic Disorder groups than for the Any Bipolar Disorder groups.

This pattern of results raises interesting questions, and there are at least two alternative explanations. First, given the same presenting symptoms, clinicians tend to diagnose certain diagnoses more than others in different countries. For example, the findings of the present study show that, in general, psychotic disorders were diagnosed more, especially in Ethiopia and Uganda, than bipolar disorder. The exception to this case may be in Kenya, where we see the highest diagnostic accuracy for bipolar disorder. This is consistent with the pattern we see in the test–retest reliability data, where Kenya has the best test–retest reliability for bipolar disorders and Ethiopia has the best test–retest reliability for psychotic disorders. Second, although the findings could be an artifact related to biases related to the tendency to diagnose bipolar disorder in Kenya and psychotic disorder in Ethiopia and Uganda, it is also possible that the presentation of psychotic and bipolar disorders vary in different places such that the diagnoses on the MINI and SCID are more or less aligned with the local presentations of these disorders. For example, existing epidemiological findings show a higher prevalence of bipolar disorder (5.2%) than psychotic disorders (1.0%) in Kenya (Kwobah, Epstein, Mwangi, Litzelman, & Atwoli, 2017). Further, estimates of psychotic and bipolar disorder in hospital settings in Ethiopia follow the same general pattern as our findings, with 39.6% of patients diagnosed with schizophrenia compared to 29.8% diagnosed with bipolar disorder (Mamaru et al., 2021). However, additional research is needed beyond the current research base to more fully evaluate the estimates of psychotic and bipolar diagnoses in these countries.

The overarching question in any study examining validity is whether the measures, including the ‘gold standard’ (i.e., SCID), are measuring the underlying constructs they were designed to measure (Brown, 2006). Given the findings that bipolar disorder had the lowest specificity in the MINI and SCID, this points to the question of whether the MINI and SCID are fully assessing the symptoms of bipolar disorder in these countries. These differences may be related to measurement issues in which the MINI and SCID, created within the Western framework of psychiatry, may be missing or not able to discern the cultural nuances related to the expression and diagnosis of bipolar disorder in sub-Saharan Africa. It is crucial for future investigations to examine this issue more fully and to assess whether the construct of bipolar disorder, as assessed using measures developed in the global North, is missing some important part of the diagnosis of this disorder in the global South. As such, the use of qualitative approaches may be beneficial to provide a nuanced evaluation of what is being assessed well in the MINI and SCID and what is being missed (or needs to be revised to fit the context) to enhance the diagnostic assessment of bipolar disorder in diverse settings.

Finally, it is interesting to note that item-level symptom endorsement is generally higher for the MINI than the SCID. This is especially true for the endorsement of mania (see Table 3), where we see that 54.3% of the sample endorsed mania on the MINI, but only 23.4% endorsed mania on the SCID. These differences raise an interesting point related to the validity of these measures and may be related to measurement issues in the way items are assessed in structured (MINI) vs semi-structured (SCID) measures. Structured measures, such as the MINI, are generally scored based on a single yes or no item and limit prompting to enhance ease of administration, whereas semi-structured instruments, such as the SCID, use probes to obtain additional detail on symptom endorsement to determine clinical significance.

*Limitations and future directions.* As with any study, our findings must be considered in light of the weakness of our study. A key limitation of the present study is that the parent study aimed to examine the neuropsychiatric genetics of the psychotic and bipolar disorder continuum, we used the general psychotic module of the version of the MINI that only allows for a general “psychotic disorder” diagnosis. This limited our ability to make direct diagnostic comparisons of specific psychotic disorders (e.g., schizophrenia) and, instead, we were required to make comparisons at the overall “any psychotic disorder” level. Future research would benefit from examining the validity and reliability of the MINI for Psychotic Disorder Studies module, which provides a more nuanced approach, allowing for the diagnosis of specific psychotic disorders (e.g., schizoaffective disorder). Further, given the focus on sub-Saharan Africa, the findings are limited in that they may have limited generalizability to other regions of the world. As such, the validity and reliability of the MINI and SCID must be assessed in other LMICs to better understand the ability of measures developed in HICs to provide valid and reliable diagnoses across the globe. Finally, this study was conducted as a substudy of the larger NeuroGAP-Psychosis parent study. As such, participants involved in the present study were recruited directly from the parent study, which could have biased the results.

Despite these limitations, there are several strengths that outweigh the weaknesses. In particular, our study utilized a large sample across three countries in sub-Saharan Africa (i.e., Ethiopia, Kenya, and Uganda) and assessed both validity and reliability of the MINI and SCID in these countries, thereby adding to the nascent literature of the psychometric properties of the MINI and SCID in diverse settings. We are also the first to demonstrate the differential pattern of diagnostic specificity in psychotic and bipolar disorders by country when using the MINI-7 and SCID.

As with any research study, it is important for future research to extend the findings of the present study, incorporating cultural nuances not captured in the present assessment. Assessing the diagnostic validity of measurement tools is an iterative process that benefits from quantitative and qualitative studies. Further exploration of the validity and reliability of the MINI and SCID in psychosis and other mental health disorders in sub-Saharan Africa and other diverse settings would be especially beneficial in learning about the use of these clinical interviews in diverse settings. Further, it will be important to assess the diagnostic reliability of the MINI-7 using the MINI for Psychotic Disorders Studies, which allows for the diagnosis of more psychotic disorders than the version used in the present study. The MINI only allows for the diagnosis of a general ‘psychotic disorder’ diagnosis rather than a more nuanced diagnostic approach, allowing for specific psychotic disorder diagnoses (e.g., schizophrenia) as obtained in the SCID. As this body of literature continues to grow, we will learn more about the expression of mental health disorders in diverse settings and, over time, be able to better evaluate how the current nosology system maps onto the expression of these diagnoses or whether our nosology system should be altered to better account for variations observed in diverse settings and cultural contexts across the globe.
